# Supplementary material for: Online and Face-to-Face Mat Pilates Training for Long COVID-19 Patients: A Randomized Controlled Trial on Health Outcomes
Source: Int J Environ Res Public Health. 2024 Oct 19;21(10):1385. doi: 10.3390/ijerph21101385 (PMC11506963; doi:10.3390/ijerph21101385)
Supplement: Supplementary file 1 [file ijerph-21-01385-s001.zip › ijerph-3253346-supplementary.pdf]

Supplementary Material

Supplementary **table S1**. Pilates exercise program – Beginner Level

Exercise	Description - Beginner Level
**Warm up**	
**The Elefant**	In a six-point stance with hands, knees, and feet in dorsiflexion. Performing joint alignment of the shoulders with the wrists and knees with the hips. The hip should be slightly abducted and in a neutral position. To prepare for the movement, one should inhale in a neutral position, and exhale to perform the spinal flexion movement, starting from the lumbar spine to the cervical spine, prioritizing spinal mobility by extending the hips and knees, bringing the heels to the ground, and reaching the hands towards the heels. Inhale again to return to the position with the hands on the ground and the initial stance.
**Mermaid**	Sitting with the hip flexed in external rotation, with one knee flexed in front of the body and the other laterally behind. One hand should be supported on the ground laterally to the body, with the shoulder slightly abducted and the elbow flexed. The other hand should be positioned laterally with the shoulder abducted, with a slight elbow flexion. To prepare for the movement, inhale in a neutral position and exhale slowly while laterally flexing the trunk towards the ground with the arm slightly flexed and extended overhead. Then, inhale again and exhale while returning to the initial position.
**The Cat**	In a six-point stance with hands, knees, and feet in plantar flexion supported on the ground. Ensure joint alignment of the shoulders with the wrists and the knees with the hips. The hip should be slightly abducted and in a neutral position. To prepare for the movement, inhale in a neutral position and exhale slowly while performing spinal flexion (lumbar, cervical, and thoracic) progressively, prioritizing vertebral mobility. Then, inhale again and exhale while performing spinal extension.
**Training A**	
**Roll up**	Seated position with knees extended, legs slightly apart, spine upright, arms stretched forward aligned with the shoulders, and feet in dorsiflexion. Take a deep breath, then exhale and lean the torso forward as if reaching towards the toes with the hands. Keep the head aligned with the arms throughout the movement.
**Swimming**	In prone position, with legs extended and feet in plantar position. The arms are crossed near the head. Lift both lower limbs off the ground and perform the movement by further raising one leg, alternating the movement of the limbs, similar to a scissor motion. After one cycle of exhalation and inhalation, one repetition is counted, and so on.
**The Hundred**	Supine position, with hips and knees flexed at 90 degrees. Arms extended and shoulders flexed at 90 degrees. Hold the position and swing the extended arms up and down in short movements, five movements inhaling and 5 movements exhaling, until reaching 100.
**The Swan dive**	Prone position, legs extended and feet in plantar flexion, shoulders abducted, elbows flexed and aligned with the head. Inhale in this position and exhale while

	extending the elbows and simultaneously extending the spine. Inhale while returning to the initial position.
**One leg circle (right / left)**	In supine position with one leg flexed and the other extended in plantar flexion, perform a complete hip circumduction clockwise, with an amplitude that does not cause instability, while inhaling and exhaling. The opposite leg remains flexed.
**The Side Kick (right / left)**	In lateral decubitus with one upper limb supporting the head and the other in contact with the ground. The leg that will be supported on the ground remains flexed, while the other is extended with plantar flexion. Keep the hip abducted and move the leg forward and backward without bending the knee, while minimizing movement of the trunk.
**The Leg Pull Front**	Starting position in four supports, wrists aligned with the shoulders and knees aligned with the hips and back in a neutral position. When starting the movement, remove one of your hands from the ground, stretching your arms forward and at the same time raising the leg opposite the arm that will be extended. Perform one repetition, return to the starting position and alternate sides.
**The Teaser**	In a seated position with knees bent, feet resting on the ground, and hands supporting behind the knees, lean slightly back. Inhale, preparing for the movement, and exhale as you lift one foot off the ground until the knee is fully extended. Return to the initial position and perform the movement with the opposite leg. Remember to keep the spine upright throughout the entire movement.
**The Double Leg Strech**	In supine position, knees bent, feet and back supported on the ground. Arms are spread apart and parallel to the trunk, but not touching the floor. Inhale and hold the breath, then exhale as you move. While exhaling, lift your feet off the ground and bring your legs and hands towards your feet, positioning them under the ankles and the knees under your chest. Return to the initial position and repeat until completing the set.
**Plank**	Starting position in four supports, wrists aligned with the shoulders, knees aligned, feet in dorsiflexion and with the hips and back in a neutral position. When starting the exercise, slightly remove your knees from the ground and maintain isometry until you complete the repetitions. When you inhale and exhale completely, this refers to one repetition.
**Training B**	
**The Leg Pull**	Seated with palms and feet supported on the ground and knees flexed. Maintain shoulders hyperextended in internal rotation. Inhale and exhale while lifting the hips, fully extending the body so that the knees are aligned with the hips and shoulders. Return to the initial position by lowering the hips back to the ground.
**The One Leg Stretch**	Starting position lying on your back in dorsal decubitus, with your back and head against the floor. One leg should be extended while the other is flexed with the knees close to the chest and both feet in plantar flexion. Hold one leg only, keeping the hand on the same side in contact with the ankle and the hand of the contralateral upper limb in contact with the knee. The contralateral lower limb should have hip flexion at 45 degrees and the knee extended. Inhale and switch the lower limbs' position, repeating the switches successively until the end of the exercise.

**The Push Up 1**	Standing in an orthostatic position. Inhale to prepare for the movement and exhale while flexing the cervical, thoracic, and lumbar spine progressively until the hands touch the ground. Walk your hands on the ground until reaching the plank position and return to the initial position.
**The Push Up 2**	In prone position, hands supported on the ground slightly wider than shoulder-width apart and directly beneath the shoulders vertically, with fingers pointing forward, and knees resting on the ground. Lift the body until the chest is away from the floor, without flexing the hips or lifting the knees off the ground. Return to the starting position and repeat the process.
**The Bicycle**	In supine position with the spine supported on the ground and hands behind the head. Hips and knees should be resting on the ground. Slowly lift the head and shoulders off the floor. Inhale slowly and exhale while flexing the torso, trying to bring the elbow of the opposite side towards the knee. Return to the initial position and perform on the opposite side.
**The Side Bend**	In lateral decubitus, flex your knees to 90 degrees and hips to 45 degrees, keeping them in contact with the ground. Support one hand on your waist and keep the shoulder abducted as needed to support body weight, maintaining alignment of the spine. During the movement, lift your hip off the ground and lower it slowly back to the initial position. After completing the repetitions, perform on the opposite side. Inhale and exhale slowly during the movement.
**The Saw**	Seated, with a neutral spine, knees extended, and legs in approximately 45° abduction. Keep the arms abducted with elbows extended, legs supported on the ground, and feet in dorsiflexion. Inhale and rotate the spine to the right. Exhale while flexing the spine forward, attempting to reach the toes with the left hand. Inhale to prepare for the movement and exhale while returning to the initial position. Repeat for both sides.
**The Side Kick Kneeling**	In lateral decubitus with support on the knee and hand. For the supporting side: keep the shoulder abducted and the elbow extended, the hip should be abducted, and the knee flexed. For the upper side: support the hand on the nape keeping the shoulder abducted and the elbow flexed, at the same time maintain the hip in an orthostatic position with the knee extended and the foot in plantar flexion in contact with the ground. Inhale and exhale while abducting the hip on the upper side, keeping the knee extended. After completing the repetitions, repeat for the contralateral segment.
**The Shoulder Bridge**	In supine position, with feet supported on the ground, hips and knees flexed, and upper limbs in an orthostatic position. Inhale in the initial position and exhale while extending the hips to neutral position, then return to the initial position. During the movement, it is important to exert force with the heels.
**The Jack Knife**	In supine position, with upper limbs alongside the body supported on the ground, hips adducted, and knees flexed at 90 degrees with heels together and feet in plantar flexion. Inhale while stationary and exhale while extending the legs, then inhale while stationary and exhale while returning to the initial position.

**Plank**	Starting position in four supports, wrists aligned with the shoulders, knees aligned, feet in dorsiflexion and with the hips and back in a neutral position. When starting the exercise, slightly remove your knees from the ground and maintain isometry until you complete the repetitions. When you inhale and exhale completely, this refers to one repetition.
**Cooling down**	
**Single Straigth Leg Stretch Adapted**	Supine with one leg flexed, foot supported on the ground, and the other extended at 90 degrees, with arms extended beside the trunk. Inhale and exhale while extending the knee simultaneously with flexing the hip to its maximum range. Inhale, maintaining the stretching position, and exhale while returning to the initial position.
**The Rocking Adapted**	Prone position, arms and legs extended along the body, hips in external rotation keeping the heels together and feet in plantar flexion. Inhale while attempting to isometrically extend the knees.
**Cat Stretch Adapted**	In a six-point stance with hands, knees, and feet in plantar flexion. Perform joint alignment of the shoulders with the wrists and the knees with the hips. The hip should be slightly abducted and in a neutral position. Inhale to prepare for the movement and exhale while performing flexion of the cervical, thoracic, and lumbar spine. Prioritize mobilization of the vertebral column, returning to the initial position.

Supplementary **table S2**. Pilates exercise program – Intermediate Level

Exercise	Description - Intermediate Level
**Warm up**	
**The Elefant**	In a six-point stance with hands, knees, and feet in dorsiflexion. Performing joint alignment of the shoulders with the wrists and knees with the hips. The hip should be slightly abducted and in a neutral position. To prepare for the movement, one should inhale in a neutral position, and exhale to perform the spinal flexion movement, starting from the lumbar spine to the cervical spine, prioritizing spinal mobility by extending the hips and knees, bringing the heels to the ground, and reaching the hands towards the heels. Inhale again to return to the position with the hands on the ground and the initial stance.
**Mermaid**	Sitting with the hip flexed in external rotation, with one knee flexed in front of the body and the other laterally behind. One hand should be supported on the ground laterally to the body, with the shoulder slightly abducted and the elbow flexed. The other hand should be positioned laterally with the shoulder abducted, with a slight elbow flexion. To prepare for the movement, inhale in a neutral position and exhale slowly while laterally flexing the trunk towards the ground with the arm slightly flexed and extended overhead. Then, inhale again and exhale while returning to the initial position.
**The Cat**	In a six-point stance with hands, knees, and feet in plantar flexion supported on the ground. Ensure joint alignment of the shoulders with the wrists and the knees with the hips. The hip should be slightly abducted and in a neutral position. To prepare for the movement, inhale in a neutral position and exhale slowly while performing spinal flexion (lumbar, cervical, and thoracic) progressively, prioritizing vertebral mobility. Then, inhale again and exhale while performing spinal extension.
**Training A**	
**Roll up**	Lying position with legs flexed and spread at hip-width, feet in dorsiflexion. Take a deep breath, then extend the legs and lift the trunk as if the hands were to touch the toes. Keep the head aligned with the arms during the movement, return to the initial position, and perform the movement again.
**Swimming**	In prone position, with legs extended and feet in plantar flexion. The arms are stretched forward with palms facing down. Lift the upper and lower limbs off the ground and perform the movement by alternating the motion of the limbs. After one cycle of exhalation and inhalation, count one repetition, and so on.
**The Hundred**	In supine position with legs stretched upward and feet in plantar flexion. Arms extended and shoulders flexed at 90 degrees. Hold the position and swing the

	extended arms up and down in short movements, five movements inhaling and five movements exhaling, until reaching a total of 100 repetitions.
**The Swan dive**	In prone position, legs extended, feet in plantar flexion, elbows flexed, and shoulders abducted, with hands supporting in front of the head. Inhale in this position and exhale while extending the elbows and simultaneously extending the spine. Inhale while returning to the initial position.
**One leg circle (right / left)**	In supine position, lying flat on the back, with legs extended on the ground. Inhale and exhale while lifting one leg up to 90 degrees from the ground. Then, inhale and exhale while performing a half circle of the hip that is flexed in the counterclockwise direction. Try to maintain the hips always in slight external rotation, especially the moving lower limb.
**The Side Kick (right / left)**	In a lateral position with one upper limb supporting the head and the other in contact with the ground, and the bottom leg extended. Keep the upper leg abducted with the knee extended. Inhale, and upon exhaling, move the upper leg forward and backward, avoiding moving the trunk as much as possible.
**The Leg Pull Front**	In six supports, with hands aligned with the shoulders, knees aligned with the hips, and feet resting on the ground. Inhale and exhale while performing leg hyperextension, keeping the foot in plantar flexion, and at the same time, extend the arm on the opposite side forward above the head. Perform all repetitions on one side before switching to the other side.
**The Teaser**	Sitting with the upper limbs supported behind the knee, legs bent with feet on the ground, and the trunk slightly leaning backward. Inhale, prepare for the movement, and exhale while lifting both feet off the ground until the knees are fully extended. Remember to keep the spine erect throughout the movement.
**The Double Leg Strech**	In supine position with the back supported on the ground and legs bent at 90 degrees. Hands are placed beside the hips. Inhale, and upon exhaling, extend the legs upward, reaching a 90-degree angle with the ground.
**Plank**	In four supports, with hands placed in line with the shoulders and legs extended, supported on the toes in dorsiflexion. Inhale and lift the body, keeping the trunk and legs aligned. Control the breath, maintaining abdominal contraction in isometric hold throughout.
**Training B**	
**The Leg Pull**	Sitting with the palms of the hands supported, aligned with the shoulders, and legs extended. Keep the shoulders hyperextended in internal rotation. Inhale and exhale while lifting the hips until they are aligned with the legs and spine, then lower slowly.
**The One Leg Stretch**	Lying on your back with legs extended and slightly elevated. Inhale, and upon exhaling, perform full knee flexion with the hand on the same side as the leg, trying

	to reach the knee, while the hand on the opposite side reaches for the knee as well. Alternate legs without placing them on the ground.
**The Push Up**	Standing in an upright position. Inhale to prepare for the movement, and exhale while progressively flexing the cervical, thoracic, and lumbar spine until the hands touch the ground. Walk the hands on the ground until reaching the plank position, then support the knees on the ground and perform a push-up until the chest touches the ground, then return to the initial position.
**The Bicycle**	Lying on your back, keep your knees bent at a 90-degree angle and your spine supported on the ground. Inhale, and upon exhaling, extend one leg while flexing the contralateral leg. Alternate legs while aiming for the greatest range of motion.
**The Side Bend**	In a side-lying position, flex the knee that is supported on the ground and extend the other leg. The forearm should be supported on the ground, aligned with the shoulder, with the elbow flexed. The contralateral hand will be placed on the waist. Inhale and perform the movement by lifting the hip until the body is aligned, then lower slowly. After completing one side, repeat on the other side.
**The Saw**	Seated, with a neutral spine, knees extended, and legs at a 60-degree angle. Keep the arms abducted with elbows extended and feet supported on the ground. Inhale and rotate the spine to the right. Exhale while flexing the spine forward, trying to reach the toes with the left hand. Inhale to prepare for the movement and exhale while returning to the initial position. Repeat for both sides.
**The Side Kick Kneeling**	In a lateral position with the knee flexed and the hand aligned with the shoulder supported on the ground. The contralateral hand is supported on the waist and the upper leg extended. Keep the hip elevated isometrically throughout the entire movement. Inhale and upon exhaling, perform abduction of the extended leg and return it to the ground. After completing the repetitions on one side, switch sides. Remember to keep the abdomen contracted throughout the movement to avoid trunk movement.
**The Shoulder Bridge**	In supine position, with feet on the ground, knees bent, and upper limbs in an upright position. Inhale in the initial position and exhale while performing full extension of one leg and maintaining isometric contraction of that leg. Keep the foot in plantar flexion. Inhale again and upon exhaling, raise and lower the hip from the ground. Complete all repetitions and repeat for the contralateral segment.
**The Jack Knife**	In supine position, with the upper limbs along the body supported on the ground and the knees bent at 90 degrees. Inhale and exhale while extending the knees forward without touching the legs to the ground. Contract the abdomen to prevent trunk movement.
**Plank**	In four supports, with forearms supported in line with the shoulders and legs extended, supported on the toes in dorsiflexion. Maintain this isometric position throughout the exercise. Control your breathing, keeping the abdomen contracted.
**Cooling down**	

**Single Straigth Leg Stretch Adapted**	Supine with one leg flexed, foot supported on the ground, and the other extended at 90 degrees, with arms extended beside the trunk. Inhale and exhale while extending the knee simultaneously with flexing the hip to its maximum range. Inhale, maintaining the stretching position, and exhale while returning to the initial position.
**The Rocking Adapted**	Prone position, arms and legs extended along the body, hips in external rotation keeping the heels together and feet in plantar flexion. Inhale while attempting to isometrically extend the knees.
**Cat Stretch Adapted**	In a six-point stance with hands, knees, and feet in plantar flexion. Perform joint alignment of the shoulders with the wrists and the knees with the hips. The hip should be slightly abducted and in a neutral position. Inhale to prepare for the movement and exhale while performing flexion of the cervical, thoracic, and lumbar spine. Prioritize mobilization of the vertebral column, returning to the initial position.

Supplementary **table S3**. Pilates exercise program – Advanced Level

Exercise	Description - Advanced Level
**Warm up**	
**The Elefant**	In a six-point stance with hands, knees, and feet in dorsiflexion. Performing joint alignment of the shoulders with the wrists and knees with the hips. The hip should be slightly abducted and in a neutral position. To prepare for the movement, one should inhale in a neutral position, and exhale to perform the spinal flexion movement, starting from the lumbar spine to the cervical spine, prioritizing spinal mobility by extending the hips and knees, bringing the heels to the ground, and reaching the hands towards the heels. Inhale again to return to the position with the hands on the ground and the initial stance.
	Sitting with the hip flexed in external rotation, with one knee flexed in front of the body and the other laterally behind. One hand should be supported on the ground

**Mermaid**	laterally to the body, with the shoulder slightly abducted and the elbow flexed. The other hand should be positioned laterally with the shoulder abducted, with a slight elbow flexion. To prepare for the movement, inhale in a neutral position and exhale slowly while laterally flexing the trunk towards the ground with the arm slightly flexed and extended overhead. Then, inhale again and exhale while returning to the initial position.
**The Cat**	In a six-point stance with hands, knees, and feet in plantar flexion supported on the ground. Ensure joint alignment of the shoulders with the wrists and the knees with the hips. The hip should be slightly abducted and in a neutral position. To prepare for the movement, inhale in a neutral position and exhale slowly while performing spinal flexion (lumbar, cervical, and thoracic) progressively, prioritizing vertebral mobility. Then, inhale again and exhale while performing spinal extension.
**Training A**	
**Roll up**	In supine position, with upper limbs in an upright position. Keep the hips externally rotated with heels together. Inhale while raising and flexing the shoulders up to 90 degrees, exhale starting the movement with cervical flexion. Then, continue the movement with flexion of the thoracic spine until maximum flexion; the upper limbs should be in front in line with the ears. Inhale and exhale while extending the spinal column starting from the sacral region, then moving to the lumbar and thoracic region, mobilizing the spine until returning to the initial position.
**Swimming**	In prone position, with arms and legs extended along the body and hips externally rotated, keeping the heels together. Inhale, further flexing the shoulders and hyperextending the hips. Perform the movement by flexing one shoulder without touching the segment to the ground, while simultaneously flexing the contralateral hip without it touching the ground either. Perform this movement alternately, always in a crossed pattern.
**The Hundred**	In supine position with legs diagonally positioned, feet in plantar flexion, and torso slightly elevated. Arms extended and shoulders flexed at 90 degrees. Maintain the position and swing the extended arms up and down in short movements, five movements inhaling and five movements exhaling, until completing a total of 100 repetitions.
**The Swan dive**	In prone position, legs extended, feet in plantar flexion, elbows flexed, and shoulders abducted, place your hands on the ground beside your face. Inhale in this position and exhale while extending the elbows and simultaneously extending the spine. Inhale while returning to the initial position.
**One leg circle (right / left)**	In supine position with legs upright. Inhale and exhale while flexing one hip up to 90 degrees with the knee extended. Then, inhale and perform a half circle motion of the flexed hip in one direction, and exhale while completing the half circle motion. Maintain a slight external rotation of the hips throughout, especially in the moving lower limb.
**The Side Kick (right / left)**	In lateral decubitus with one upper limb supporting the head and the other in contact with the ground. Keep the right hip abducted and the knee extended. Inhale, and upon exhaling, move the legs alternately forward and backward, making sure to minimize movement of the trunk as much as possible.

**The Leg Pull Front**	In plank position, shoulders flexed at 90 degrees with palms of the hands supported on the ground. Inhale and exhale while performing hyperextension of the right hip, keeping the foot in plantar flexion. Exhale while executing plantar flexion of the supporting foot, and then, during inhalation, return to dorsiflexion, maintaining hyperextension of the right hip. Exhale again while returning the right hip to the initial position. Repeat the same movement with the left lower limb, alternating with each repetition.
**The Teaser**	In supine position with the upper limbs along the body. Inhale, preparing for the movement, and exhale while flexing the spine and the hips. Simultaneously, perform shoulder flexion with elbows extended. Inhale, maintaining the position isometrically, and exhale while returning to the initial position.
**The Double Leg Strech**	In supine position, maintain high flexion of the cervical and thoracic spine. The lower limbs should be in hip and knee flexion until they can be enveloped by the upper limbs. Exhale while extending the hips to 45 degrees simultaneously with full extension of the knees. At the same time, flex the shoulders with elbows extended to 135 degrees. Inhale and exhale while returning to the initial position.
**Plank**	In four supports, with forearms supported in line with the shoulders and legs extended, supported on the toes in dorsiflexion. Control your breathing, keeping the abdomen contracted in isometric hold throughout.
**Training B**	
**The Leg Pull**	Sitting with the palms of the hands supported on the ground. Keep the shoulders hyperextended in internal rotation. Inhale and exhale while extending the hip simultaneously with performing plantar flexion to place the soles of the feet on the ground. Inhale and exhale while flexing one hip, keeping the foot in plantar flexion. Inhale while returning the lower limb to the initial position.
**The One Leg Stretch**	Starting the movement from the final position of the previous exercise. Hold one leg only, with the hand of the same side in contact with the ankle and the hand of the contralateral upper limb in contact with the knee. The contralateral lower limb should be at 45 degrees hip flexion with the knee extended. Perform extension of the lumbar spine, keeping the thoracic spine high and cervical spine flexed. Inhale and switch the lower limbs' positions, repeating the exchanges successively until the end of the exercise.
**The Push Up**	Standing in an upright position. Inhale to prepare for the movement and exhale while progressively flexing the cervical, thoracic, and lumbar spine until the hands touch the ground. Walk the hands on the ground until reaching the plank position. Inhale while flexing the elbows and exhale while extending them again for three repetitions. Inhale and exhale while returning to the initial position.
**The Bicycle**	In supine position, keep the hips flexed at 90 degrees and the lumbar spine in flexion without contacting the ground, using the hands and bent elbows supported on the ground to sustain the lumbosacral region. Inhale and extend one hip while flexing the contralateral hip. The knees should flex with hip extension and extend with hip flexion throughout the alternating movement for each segment. Aim to achieve the maximum range of motion. Reverse the position of the lower limbs.

**The Side Bend**	In left lateral decubitus, bend the knees to 90 degrees and the hips to 45 degrees, keeping them in contact with the ground. Place the palm of the left hand on the ground, with the shoulder abducted as needed to support body weight. Also, the spine should be in right lateral flexion. Inhale and exhale while performing left lateral flexion of the spine, extending the knees and hips. Inhale while returning to right lateral flexion, without contacting the ground, keeping the hips and knees extended. Simultaneously, rotate the cervical spine to the right. Exhale while performing left lateral flexion of the spine to the maximum range of motion, rotating the cervical spine to the left. Inhale while maintaining the position isometrically, and exhale while returning to the initial position.
**The Saw**	Seated, with a neutral spine, legs extended, and legs in a 90-degree angle. Keep the shoulders abducted with elbows extended and feet supported on the ground. Inhale and perform rotation of the spine to the right. Exhale while flexing the spine forward, attempting to reach the toes with the left hand. Inhale to prepare for the movement and exhale while returning to the initial position. Repeat for both sides.
**The Side Kick Kneeling**	In lateral decubitus with support on the knee and hand. For the supporting side: keep the shoulder abducted and the elbow extended, the hip should be abducted, and the knee flexed. For the upper side: support the hand on the nape keeping the shoulder abducted and the elbow flexed, at the same time maintain the hip in an orthostatic position with knee extended and foot in plantar flexion in contact with the ground. Inhale and exhale while abducting the hip on the upper side, keeping the knee extended. Then, inhale while flexing the hip forward and exhale while extending it back to the previous position.
**The Shoulder Bridge**	In supine position, with feet supported on the ground, hips and knees flexed, and upper limbs in an orthostatic position. Inhale in the initial position and exhale while extending the hips to neutral position. Inhale again while extending the knee of one leg and flexing this hip to 90 degrees. Keep the foot in plantar flexion. Exhale while dorsiflexing the same foot, extending the hip, and flexing the knee back to the previous position. Repeat for the contralateral segment.
**The Jack Knife**	In supine position, with upper limbs alongside the body supported on the ground and hips adducted and externally rotated with heels together. Inhale and flex the hips to 90 degrees without interrupting the contact of the heels and external rotation. Then, flex the lumbar and thoracic spine with minimal addition to hip flexion possible. Pay attention to mobilizing the spine. Engage the arms against the ground. Exhale while contracting the glutes and try to extend the spine and hips while maintaining support over the scapular region. Inhale while returning the spine to flexion, without touching the ground, and gradually extend the spine against gravity with focus on mobilization until reaching the initial position.
**Plank**	In four supports, with your forearms resting on your shoulders and your legs stretched out, resting on your toes in dorsoflexion. Control your breathing by keeping your abdomen contracted the entire time in isometrics.
**Cooling down**	
**Single Straigth**	Supine with one leg flexed, foot supported on the ground, and the other extended at 90 degrees, with arms extended beside the trunk. Inhale and exhale while

**Leg Stretch Adapted**	extending the knee simultaneously with flexing the hip to its maximum range. Inhale, maintaining the stretching position, and exhale while returning to the initial position.
**The Rocking Adapted**	Prone position, arms and legs extended along the body, hips in external rotation keeping the heels together and feet in plantar flexion. Inhale while attempting to isometrically extend the knees.
**Cat Stretch Adapted**	In a six-point stance with hands, knees, and feet in plantar flexion. Perform joint alignment of the shoulders with the wrists and the knees with the hips. The hip should be slightly abducted and in a neutral position. Inhale to prepare for the movement and exhale while performing flexion of the cervical, thoracic, and lumbar spine. Prioritize mobilization of the vertebral column, returning to the initial position.
